# Chimeric Infectious Bursal Disease Virus-Like Particles as Potent Vaccines for Eradication of Established HPV-16 E7–Dependent Tumors

**DOI:** 10.1371/journal.pone.0052976

**Published:** 2012-12-31

**Authors:** Juan Martin Caballero, Ana Garzón, Leticia González-Cintado, Wioleta Kowalczyk, Ignacio Jimenez Torres, Gloria Calderita, Margarita Rodriguez, Virgínia Gondar, Juan Jose Bernal, Carlos Ardavín, David Andreu, Thomas Zürcher, Cayetano von Kobbe

**Affiliations:** 1 Laboratory Animal Unit, Barcelona Biomedical Research Park, Barcelona, Spain; 2 Cancer Vaccines Unit, R & D Department, Chimera Pharma S.L.U., Madrid, Spain; 3 Department of Immunology and Oncology, Centro Nacional de Biotecnología/CSIC, Madrid, Spain; 4 Department of Experimental and Health Sciences, Pompeu Fabra University, Barcelona Biomedical Research Park, Barcelona, Spain; Federal University of São Paulo, Brazil

## Abstract

Cervical cancer is caused by persistent high-risk human papillomavirus (HR-HPV) infection and represents the second most frequent gynecological malignancy in the world. The HPV-16 type accounts for up to 55% of all cervical cancers. The HPV-16 oncoproteins E6 and E7 are necessary for induction and maintenance of malignant transformation and represent tumor-specific antigens for targeted cytotoxic T lymphocyte–mediated immunotherapy. Therapeutic cancer vaccines have become a challenging area of oncology research in recent decades. Among current cancer immunotherapy strategies, virus-like particle (VLP)–based vaccines have emerged as a potent and safe approach. We generated a vaccine (VLP-E7) incorporating a long C-terminal fragment of HPV-16 E7 protein into the infectious bursal disease virus VLP and tested its therapeutic potential in HLA-A2 humanized transgenic mice grafted with TC1/A2 tumor cells. We performed a series of tumor challenge experiments demonstrating a strong immune response against already-formed tumors (complete eradication). Remarkably, therapeutic efficacy was obtained with a single dose without adjuvant and against two injections of tumor cells, indicating a potent and long-lasting immune response.

## Introduction

Cervical cancer is the second most common cancer in women worldwide, with approximately 500,000 newly diagnosed cases and 275,000 deaths each year [1], [2], [3]. Cervical intraepithelial neoplasia (CIN), the precursor to cervical cancer, is caused by human papillomavirus (HPV) infection, and HPV type 16 is responsible for up to 55% of cases.

Current therapies include surgery and/or aggressive approaches with strong side effects. No therapeutic CIN or cervical cancer vaccine has been marketed to date, and the recently approved prophylactic HPV vaccines are not expected to significantly reduce the incidence of cervical cancer in the next 20 years [4], [5], [6]. Current attempts to obtain a cure focus mainly on the development of vaccines capable of inducing a specific immune response against tumor cells. The HPV-16 oncoproteins E6 and E7 are the main inducers of malignant transformation and are therefore ideal targets for cytotoxic T lymphocyte (CTL)–mediated immunotherapy [6], [7].

Current immunotherapy strategies are based on naked peptides, tumor cells, dendritic cells, DNA and viral particles, all of which have been used as vaccines [8], [9], [10]. In the last few years, the use of virus-like particles (VLP) has proven to be a safe alternative strategy for vaccination [11]. VLPs are a highly effective type of subunit vaccine that mimic the overall immunogenic structure of virus particles but lack their infectious genetic material. These properties make VLP excellent prototypes for safe vaccines. In addition to their ability to stimulate B cell (antibody)–mediated immune responses, VLPs have also proven highly effective at stimulating CD4 proliferative and CTL responses [12].

Infectious bursal disease virus (IBDV) VLPs are based on the assembly of a single protein (VP2) into 20 trimeric clusters of VP2 with T1 symmetry and a diameter of approximately 25 nm [13], [14]. Chimeric IBDV-VLPs incorporate heterologous amino acid sequences (antigens) fused to the N and C termini of VP2 or inserted into the hypervariable loops of VP2, thus hiding the antigen inside the VLP or presenting it on the surface of the VLP [13], [14]. Expression of the recombinant IBDV-VP2 capsid protein in yeast cells (*Saccharomyces cerevisiae*) leads to the intracellular assembly of VLP, which can be isolated and purified for *in vivo* testing.

IBDV-VLPs elicit a strong antibody-mediated (B cell) immune response in chickens [15], mice [15], [16], and rabbits (J.J.B. unpublished results), thus making them excellent candidate vaccines. The combination of tumor antigens and IBDV-VLPs represents a new strategy to boost the immune response and direct it against different types of cancer. In this context, an active role for a T cell–mediated immune response (CTL response) against tumors has been reported [17].

No data are available on IBDV-VLP as a carrier of vaccines for human use, despite numerous reports on the use of other VLPs to induce both B-cell and T-cell responses [18], [19]. The aim of this work was to analyze the therapeutic efficacy of IBDV-VLP–based vaccines carrying HPV-16 E7 epitopes against tumors induced in mice.

For this purpose, we generated a vaccine incorporating a long C-terminal fragment of HPV-16 E7 protein in the IBDV-VLP (VLP-E7) and tested its efficacy in humanized MHC Class I antigen presentation transgenic C57BL/6 mice expressing the HLA-A2 allele. These mice are able to present antigens in both murine and human MHC class I molecules [20]. VLP-E7 vaccine elicited an IFN- γ -mediated response, as measured by enzyme-linked immunospot (ELISPOT) assay and, importantly, was able to completely eradicate large established tumors. The *in vivo* tumor induction model used in this work involves the subcutaneous injection of the very aggressive TC1/A2 tumor cells, which express HPV-16 E6/E7 antigens [21].

## Methods

### Mice and Cell Lines

Transgenic HLA-A2 mice express the α1, α2, and α3 domains of HLA-A2.1 [20]. Mice were housed in a pathogen-free barrier area and sacrificed in accordance with the Guidelines for Humane Endpoints for Animals Used in Biomedical Research.

The animal care standards of Barcelona Biomedical Research Park (PRBB [Parc de Recerca Biomèdica de Barcelona]) were followed. These standards comply with European legislation on the care and use of animals.

All animal experiments were performed under the experimental protocol approved by the PRBB Institutional Committee for Care and Use of Animals, and the animals were observed on a daily basis. They were sacrificed at any sign of disease other than the induced tumor and tissue samples (normal and pathological) were recovered for histological and molecular analysis.

TC1/A2 tumor cells are derived from primary lung epithelial cells (provided by Dr. TC Wu, see also [21]) that are co-transformed with the HPV-16 oncoproteins E6 and E7 and c-Ha-*ras* oncogenes and stably express the HLA-A2 complex [21]. In addition, this cell line can present T cell epitopes in both murine MHC Class I (H2D^b^) and the human HLA-A2, thus making it an ideal vehicle for the study of immunotherapy against HPV-induced cervical cancer. The TC1/A2 tumor cell line was cultured in RPMI 1640 (Sigma) supplemented with 10% heat-inactivated fetal calf serum (Hyclone), 2 mM L-glutamine (Gibco), 5 mM 2-mercaptoethanol (Gibco), and penicillin and streptomycin (100 µg/ml). Cells were incubated at 37°C in 5% CO_2_. The cells were myoplasma-free (as analyzed periodically using polymerase chain reaction [PCR]) and were tested in nude mice for the presence of other pathogens that could interfere with the experiments. Approximately 3 weeks after intra-peritoneal injection of 1×10^5^ TC1/A2 cells, the mice were sacrificed and histopathology testing ruled out the presence of pathogens.

### Peptide Antigens

E7 45–98 (AEPDRAHYNIVTFCCKCDSTLRLCVQSTHVDIRTLEDLLMGTLGIVCPICSQKP) as well as shorter E7 11–20 (CYMLDLQPETT), 49–57 (CRAHYNIVTF) and 86–93 (TLGIVCPI) sequences were made in C-terminal carboxamide form by Fmoc-based solid phase synthesis protocols [22] and purified by preparative RP-HPLC. E7 11–20 and 49–57 sequences were N-terminal elongated with a Cys (C). For all peptides, purities of 95% or higher were established by HPLC, and identities satisfactorily confirmed by MALDI-TOF MS.

### Vaccine Generation

All the constructs for IBDV-VLP yeast expression were cloned into the pESC-URA plasmid (Invitrogen). The control, **VLP-WT**, corresponded to the IBDV VP2 sequence cloned into the *EcoRI-NotI* sites of pESC-URA. To generate the **VLP-E7** construct, the HPV-16 E7 sequence was obtained from GeneArt in the pMK vector. The cDNA sequence corresponding to amino acids 45–98 of E7 was obtained by PCR and cloned into the *NotI-HindIII* sites of pESC-URA-IBDV-VP2. **VLP-E7-B** was selected from an insertion library, which was generated using random insertions of Mu transposon (Finnzymes) along the entire IBDV VP2 sequence. In a second step, the selected large HPV-16 E7 C-terminal fragment flanked by flexible linkers was inserted into the VP2 insertion library, and VLP-forming clones were selected. The VLP-E7-B construct contains the E7 sequence between the VP2 amino acids 436 and 437.

### VLP Production and Purification

VLPs were expressed in the Y499 yeast strain. The pESC-URA constructs were introduced using a basic yeast transformation protocol. After three days of growth in YNB+CSM-URA (2% galactose) culture medium, the yeasts were lysed, and the VLPs were partially purified using a standard ammonium sulfate precipitation protocol. The ammonium sulfate fractions were analyzed using sodium dodecyl polyacrylamide gel electrophoresis (SDS-PAGE) and Coomassie staining to detect VLPs, and these samples were then fractionated using fast protein liquid chromatography (AKTA purifier UPC 10 Frac-920 system, GE-Healthcare). The purified VLPs were then analyzed by negative-stain electron microscopy (2% uranyl acetate) to investigate VLP size and morphology. The VLP samples were DNA-free, as analyzed using agarose gels loaded with >1 µg of VLP and stained with SYBR green.

### Murine IFN- γ ELISPOT Assay

Transgenic mice (C57BL/6-Tg, HLA-A2.1, 1Enge/J) were immunized subcutaneously twice (days 0 and 7) with different vaccine formulations containing 50 µg/mouse/injection of VLPs (for details of each assay, see figure legends). Eight days after the last immunization, the spleens were removed and splenocytes isolated and frozen. Epitope-specific interferon gamma (IFN-γ)–secreting splenocytes were counted *ex vivo* using an ELISPOT assay (Diaclone) with CD8^+^ T-cell epitope peptides, according to the manufacturer’s protocol. IFN- γ spots were counted using an ELISPOT reader system (BIO-SYS GmbH). Results were calculated as IFN- γ –positive cells per 1×10^6^ splenocytes ± SEM.

### Therapeutic Tumor Challenge Assays

To generate tumors, 5×10^5^ TC1/A2 cells/mouse were resuspended in 200 µl of phosphate-buffered saline and injected into the right rear flank of the study mice (day 0). After inoculation, the animals were randomly distributed into different groups and labeled individually. For the therapeutic studies, mice were immunized subcutaneously after the tumor challenge (on days 5 and 12, or 9 and 16, or only on day 9) with various vaccine formulations containing 50 µg/mouse/injection of VLP (see figure legends of each therapeutic assay). Tumor growth was periodically monitored (every 5–6 days), and tumor size was measured in cm^3^. Mice were sacrificed when the tumor reached 1 cm^3^ or earlier if the tumor ulcerated or the animals showed signs of discomfort. For the re-challenge, mice were injected with TC1/A2 cells, as described above, at day 56 after the first tumor challenge.

### Statistical Analysis

Statistical analysis was done using paired t-tests. Survival was compared among groups using log-rank test in SPSS software. For each test, p value less than 0.05 and 0.001 were considered significant and very significant, respectively. Error bars represent ±s.e.m.

## Results

### Cloning of IBDV-VLP–based CIN-cervical Cancer Vaccine Candidates

**Figure 1:**
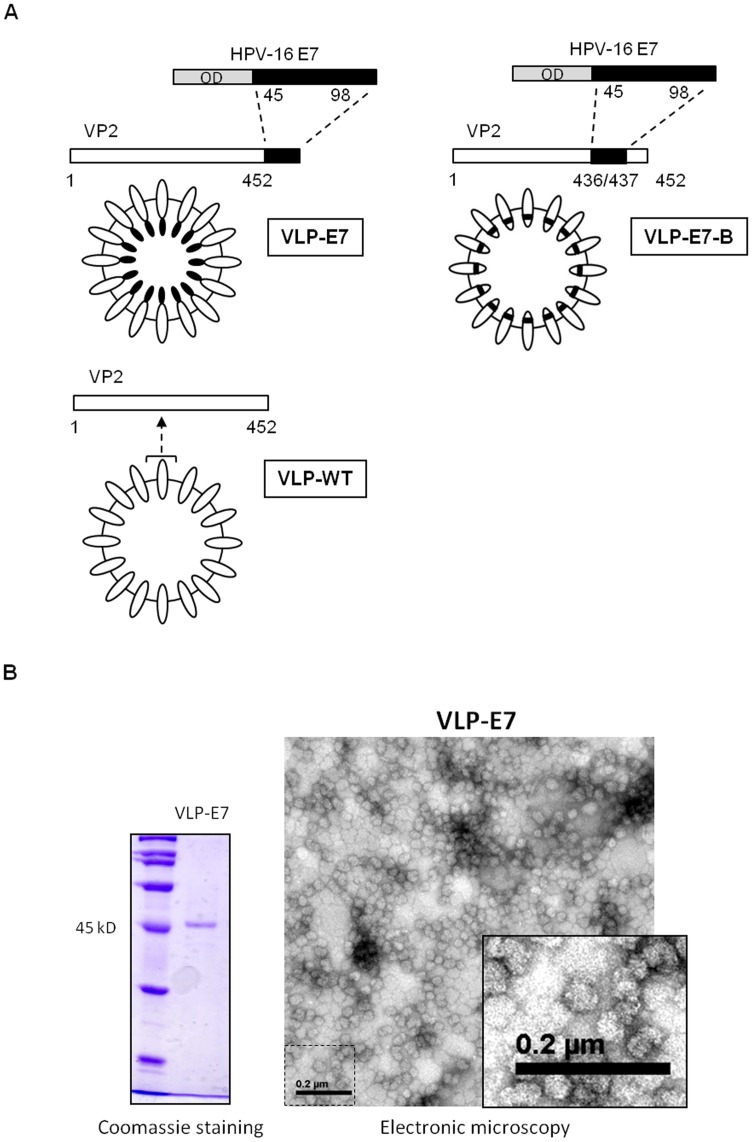
Vaccines used in this study and characterization of our vaccine candidate. **A)** IBDV VP2 is a 452-aa–long protein and is the only component of the T1 symmetry IBDV VLP (VLP-WT). A C-terminal region of HPV-16 E7 (lacking the N-terminal oncogenic domain, OD) encompassing aa 45–98 was cloned in the C-terminal part of VP2, giving rise to a chimeric VLP with the heterologous sequence inside the VLP (VLP-E7). VLP-E7-B was generated by inserting the HPV-16 E7 C-terminal region into the VP2–thus generating a chimeric VLP containing the heterologous sequence inside the VLP–and inserted into the VP2, as indicated in the figure. An illustrative drawing of each construct shows the IBDV VP2 viral capside, according to reported structural data [13] **B)** Left, the purity of VLP-E7 was analyzed by SDS-PAGE and Coomassie staining. One microgram of the vaccine was loaded onto the protein gel. Right, electron micrograph of a representative purified sample of VLP-E7 showing the virus-like structure. The bar scale (0.2 µm) is shown in the micrograph.

The IBDV VP2 protein is the only component of the T1 symmetry–based VLP (VLP-WT). This VLP contains 20 trimeric clusters of VP2 and displays hypervariable loops on its surface (Figure 1A) [13], [14]. To generate the CIN-cervical cancer candidate vaccines, one long HPV-16 E7 epitope was fused to two domains of the IBDV VP2 protein. Our first vaccine candidate (VLP-E7) contained HPV-16 E7 region 45–98, which lacks the E7 N-terminal oncogenic domain and is fused to the C terminus of the VP2. The chimeric construct expresses the E7 domain inside the VLP (Figure 1A, VLP-E7). The vaccines were expressed in yeast and purified as described in Methods. The purity of the samples was analyzed using electron microscopy and SDS-PAGE and Coomassie staining (Figure 1B). The VLP-E7 construct was approximately 45 nm in size. The presence of the E7 domain was analyzed using Western blot with specific anti-E7 antibodies (CVK, unpublished results).

The other vaccine candidate–VLP-E7-B–was generated in order to test their anti-tumor response (Figure 1A). In VLP-E7-B, the same E7 region as in the VLP-E7 construct was inserted into the amino acids 436/437 of the VP2 protein. The resulting chimeric vaccine contained the heterologous sequence inside the VLP. The localization of the E7 sequences into each construct is based on published structural data [13].

### Specific IFN- γ Secretion Induced by CIN-cervical Cancer Candidate Vaccine (ELISPOT assay) and Co-adjuvant Activity

**Figure 2:**
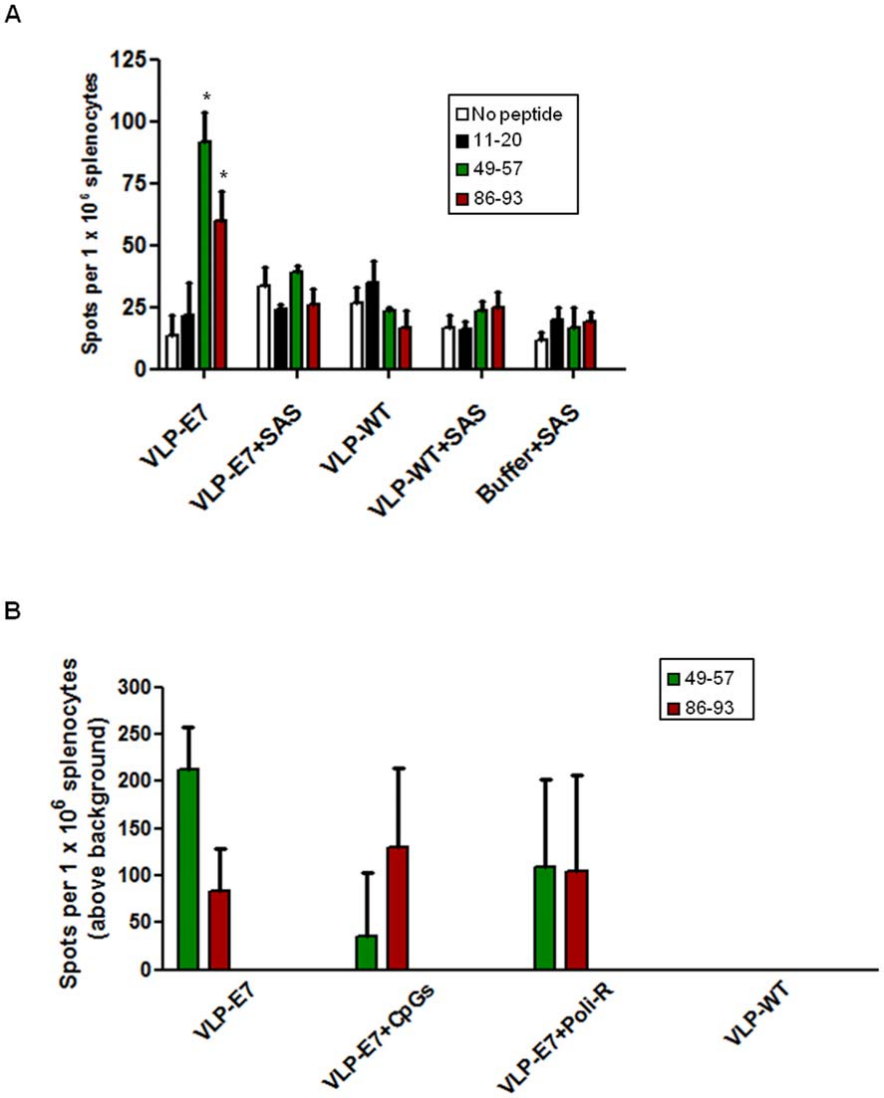
Immunization with VLP-E7 elicits E7 peptide–directed IFN- γ –secreting splenocytes. **A)** IFN- γ –secreting splenocytes from mice (n = 5) immunized twice with either 50 µg of VLP-E7 or VLP-WT or PBS, with or without Sigma Adjuvant System (SAS), were quantified *ex vivo* by ELISPOT assay. The splenocytes were pooled from each group of mice, plated, and either not stimulated (no peptide) or stimulated with control peptide (11–20) or with two E7 peptides (49–57, H2^b^ epitope and 86–93 HLA-A2 epitope). Bars represent mean ± s.e.m. of six replicates. ^*^p<0.05 compared with VLP-WT. **B)** IFN- γ –secreting splenocytes from mice (n = 5) immunized twice with either 50 µg of VLP-WT or 50 µg of VLP-E7 alone or with adjuvants (50 µg CpGs or 50 µg Poly-R) were quantified by ELISPOT assay. The splenocytes were pooled from each group of mice, plated, and stimulated as in A. The background values (no peptide and peptide control wells) were subtracted from those stimulated with the 49–57 and 86–93 E7 peptides. Bars represent mean ± s.e.m. of six replicates.

Th1 response induction is essential for an effective anti-tumor response. Therefore, we examined the capacity of VLP-E7 to elicit IFN- γ secretion when used to immunize mice. For this purpose, we quantified epitope-specific IFN- γ secretion in splenocytes harvested *ex vivo* from the mice immunized with VLP-E7 or VLP-WT or PBS alone, with or without adjuvant. For IFN- γ induction, splenocytes were treated with one E7 murine T epitope (49–57) [23], one E7 human T epitope (86–93) [24], one control E7 peptide (11–20, not included in the VLP-E7 construct, see Figure 1A), or no peptide. In the first experiment (Figure 2A), we used an oil-in-water emulsion (Sigma Adjuvant System, SAS) as an alternative to the well-known Freund water-in-oil adjuvant. SAS is derived from bacterial and mycobacterial cell wall components that generate potent Th1, antibody, and NK immune responses [25]. Interestingly, we obtained more IFN- γ –secreting splenocytes from mice immunized with VLP-E7 alone than from mice immunized with SAS adjuvant. The response obtained was clearly specific towards the E7 peptides contained in the VLP-E7 vaccine. In contrast, splenocytes from mice immunized with VLP-WT or PBS did not secrete IFN- γ above background (Figure 2A).

To investigate whether other adjuvants could boost activation of CTL by VLP-E7, we used CpGs and Poly-R. CpGs directly stimulates B cells and plasmacytoid DCs, thereby promoting Th1, antibody, and CTL immune responses. Poly-R, an arginine polymer mimicking viral RNA, promotes Th1 and CTL responses [25]. To obtain a higher specific response than the previous assay, we increased the number of splenocytes per well with respect to the first experiment and subtracted the values of the controls (no peptide and control peptide; background) from the E7-specific values. As shown in Figure 2B, neither CpGs nor Poly-R induced more IFN- γ –secreting splenocytes than those from mice immunized with VLP-E7 alone. The differences between the three groups were not significant, although they were clearly above background. In contrast, splenocytes from mice immunized with VLP-WT did not provide a specific response above background. These results demonstrate that IFN- γ –secreting immune response obtained with VLP-E7 alone was at the same level as VLP-E7+CpGs or VLP-E7+Poly-R and clearly better than VLP-E7+SAS, suggesting a co-adjuvant activity of the IBDV-VLP.

In order to test this possibility, we performed a series of dendritic cell (DC) maturation experiments, using both human and murine DCs (hDCs and mDCs, respectively)(Figure S1A). As shown in Figure S1B, IBDV-VLPs were able to induce a significant maturation of inmature hDCs, as determined/confirmed by the upregulation of CD80 and CD86. Induction with the cocktail of cytokines as positive control was (as expected) the most efficient (82%), and the addition of IBDV-VLP enhanced this process up to ∼90%.

We sought to determine whether this effect were similar with mDCs. For this purpose we performed maturation experiments with monocyte-derived mDCs. As shown in Figure S1C, IBDV-VLPs were as effective as LPS in inducing the mDCs maturation, as determined/confirmed by the upregulation of CD80, CD40 and MHC-II.

Cytokine production by mature DCs is considered to be crucial for the induction of T helper cell polarization. IL-12 and IL-6 have been considered key cytokines in promoting the generation and survival of antigen-specific CTL response [26], [27]. These data prompted us to investigate the expression profile of these cytokines in mature mDCs upon activation with either LPS or IBDV-VLP. As shown in Figure S2, mature mDCs stimulated by VLP secreted IL-12p70 and IL-6, almost as efficiently as mature mDCs stimulated by LPS. These *in vitro* results support the *in vivo* ELISPOT data, suggesting the induction of aTh1-response activation by IBDV-VLP.

The efficiency of DC activation induced by IBDV-VLPs, suggest the existence of receptors on the DC surface able to bind the VLP. To demonstrate this possibility we performed a binding assay either at 4°C or 37°C (Figure S3A). The IBDV-VLPs on the DC surface were detected by a specific mAb anti-VP2 and subsequent FACS analysis (see Figures S3A and S3B). The results clearly show a stable binding to the DC surface prior the internalization process (39% *vs* 8%, respectively).

Altogether these results demonstrate the following: i) our vaccine candidate elicited specific IFN- γ secretion from splenocytes stimulated with HPV-16 E7-specific epitopes; ii) IBDV-VLPs have co-adjuvant activity, by inducing *in vitro* maturation of human and murine DCs. iii) IBDV-VLPs bind to hDC surface receptors prior internalization and subsequent DC maturation processes.

Thus, once the immunization protocol was established, we performed the *in vivo* tumor challenges in the absence of adjuvants.

### Therapeutic Effectiveness of CIN-cervical Cancer Candidate Vaccine

**Figure 3:**
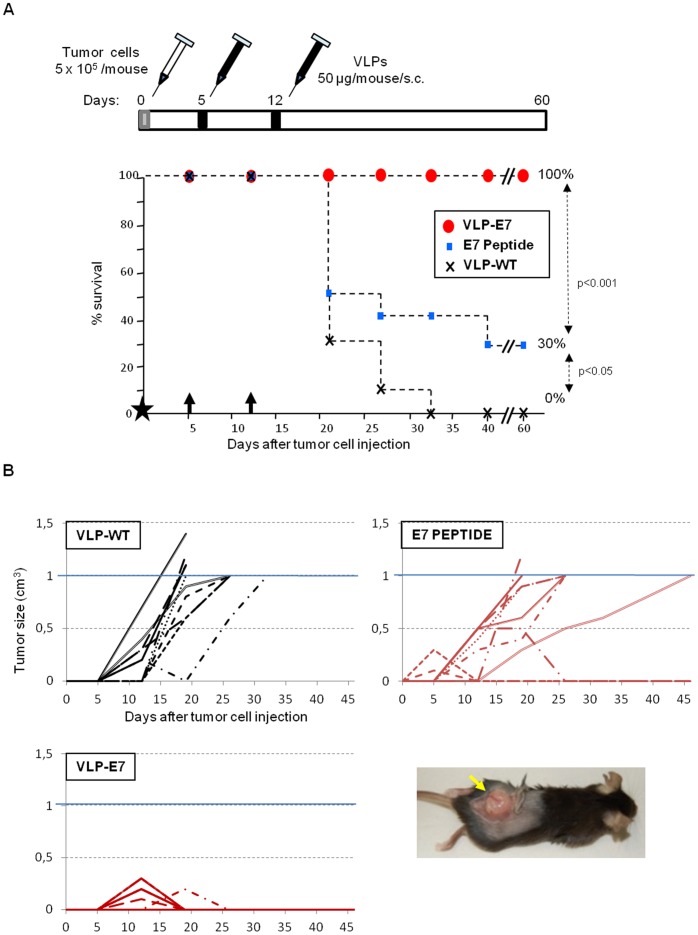
Therapeutic tumor challenge I. **A)** Top, vaccination schedule. Bottom, tumor survival data (n = 10). Black star corresponds to day 0, when tumor cells were inoculated. Black arrows indicate the vaccination days (5 and 12). Symbols are as indicated in the square. **B)** Evolution of tumor size. Values shown are from individual mice in each group, as indicated in each panel. Animals were sacrificed when tumors reached 1 cm^3^ (blue line). A representative tumor-bearing control mouse (necropsy) is shown in the photograph.

In order to evaluate the therapeutic efficacy of the CIN-cervical cancer candidate vaccine VLP-E7, mice were challenged with TC1/A2 tumor cells. At days 5 and 12 after the tumor challenge, three groups of mice (n = 10 per group) received a subcutaneous injection of one of the following vaccines: control VLP-WT, VLP-E7 vaccine, or the E7 domain included in the VLP-E7 vaccine as a naked peptide (HPV-16 E7 CT). The mice were periodically monitored for survival (Figure 3A), tumor development (Figure 3B), and general health parameters (weight, data not shown). The optimal dose of the vaccines (50 µg/mouse/injection) was based on a previous dose-dependent tumor challenge efficacy experiment (data not shown) and HPV-VLP–related vaccine data [28].

Twenty-five of the 30 animals included in the study (83%) developed tumors ranging in size from 0.2 cm^3^ to 1 cm^3^ (Figure 3B). After 20 days, all of the mice that received the control vaccine (VLP-WT) developed tumors, which reached a volume of 1 cm^3^ between day 20 and day 32 post-challenge. These findings highlight the aggressiveness of the TC1/A2 cells.

Of note, 5 out of 10 VLP-E7–vaccinated mice developed tumors (0.1–0.3 cm^3^), which were completely rejected in all cases, leaving the mice tumor-free at the end of the study (day 60) (Figure 3A and 3B). Interestingly, mice vaccinated with HPV-16 E7 C-terminal naked peptide showed limited tumor protection, and only 30% survived without tumors (Figure 3A and 3B), despite a five-fold molar excess with respect to the E7 peptide in our VLP-E7 candidate. The three surviving animals in this group rejected their tumors, including a well-established tumor of 0.5 cm^3^ (Figure 3B).

The study finished 60 days after inoculation, and the remaining mice were sacrificed. Our results demonstrate the therapeutic efficacy of the VLP-E7 vaccine against a very aggressive subcutaneously induced tumor and show a significant advantage of VLP-based vaccination technology over naked peptide in an adjuvant-free setting.

### Therapeutic Effectiveness in a More Stringent Vaccination Schedule and Comparison of Two CIN-cervical Cancer Candidate Vaccines

To test whether the site of the E7 antigen in VLPs affected the immune response against established tumors and, thus, efficacy, we cloned a new candidate: VLP-E7-B (Figure 1A). VLP-E7-B displays the 45–98 HPV-16 E7 epitope inserted into the IBDV VP2.

To evaluate the therapeutic efficacy of two CIN-cervical cancer candidate vaccines, the study mice were challenged with TC1/A2 tumor cells and randomly distributed into 3 groups (n = 9). In the previous tumor challenge (Figure 3), no large tumors (over 0.5 cm^3^) developed in VLP-E7–vaccinated animals, thanks to the potent immune response induced by the first vaccination at day 5 after injection of the tumor cells. In order to test therapeutic efficacy against well-established tumors, vaccinations were delayed 4 days to obtain tumors over 0.5 cm^3^.

At days 9 and 16 post-challenge, mice received a subcutaneous injection of VLP-WT (n = 9), VLP-E7 (n = 9), or VLP-E7-B (n = 9) and were periodically monitored for tumor development.

**Figure 4:**
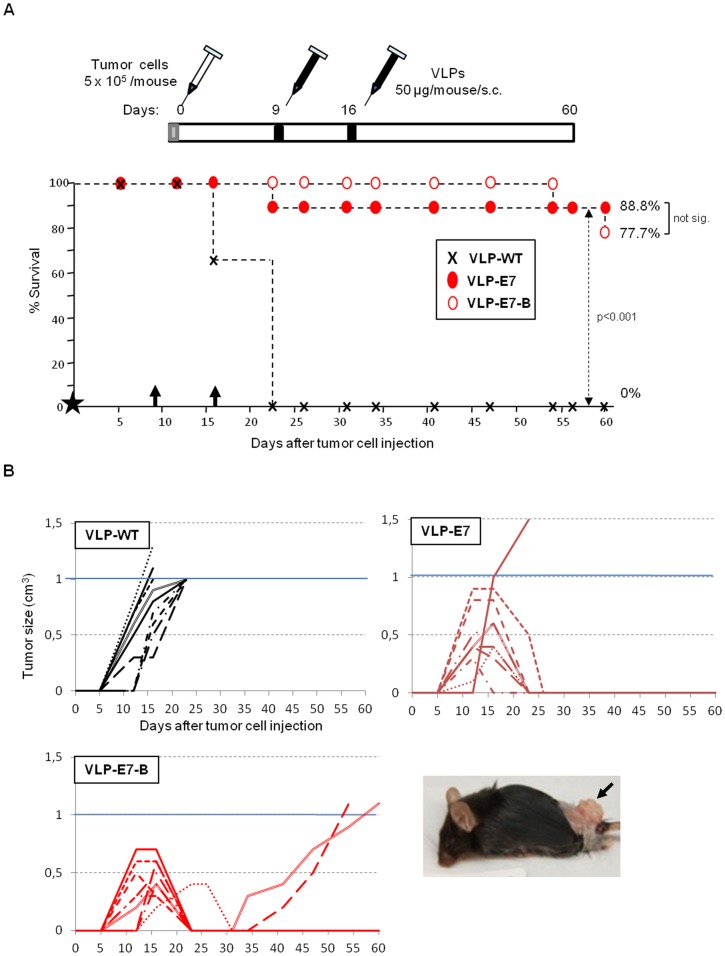
Therapeutic tumor challenge II. **A)** Top, vaccination schedule. Bottom; tumor survival data (n = 9). Black star corresponds to day 0, when tumor cells were inoculated into the mice. Black arrows indicate the vaccination days (9 and 16). Symbols are as indicated in the square. **B)** Evolution of tumor size. Values shown are from individual mice in each group, as indicated in each panel. Animals were sacrificed when tumors reached 1 cm^3^ (blue line). A representative tumor-bearing control mouse (necropsy) is shown in the photograph.

All the animals developed tumors 12–16 days after inoculation (Figure 4B). The tumors ranged in size from 0.3 to 1 cm^3^. All the mice that received the control vaccine (VLP-WT) rapidly developed tumors over 1 cm^3^ and were sacrificed by day 23; however, only one of the 18 VLP-E7–vaccinated animals had to be sacrificed by day 23 (Figure 4B, VLP-E7 group).

During the course of the experiment, two additional VLP-E7–vaccinated mice (from the VLP-E7-B group) maintained or developed slow-growing tumors during the later stage of the study (day 23-day 60) (Figure 4B). These early developed tumors of 0.4 cm^3^ and 0.6 cm^3^ were eradicated by day 23, although the tumors reappeared around 10 days later and grew to more than 1 cm^3^ between day 54 and day 60 (Figure 4B).

It is noteworthy that large tumors regressed in the VLP-E7–vaccinated animals. In some cases, tumors of 0.5 to 0.9 cm^3^ were completely eradicated, and the animals remained tumor-free until the end of the study at 60 days after inoculation (8 mice from the VLP-E7 and VLP-E7-B groups). The survival results (Figure 4A) were as follows: VLP-E7–vaccinated mice, 88.8% (8/9); and VLP-E7-B–vaccinated, 77.7% (7/9). In contrast, none of the VLP-WT–vaccinated mice remained alive beyond 23 days.

No significant differences were observed between the two vaccines tested (VLP-E7 and VLP-E7-B, p>0.05), indicating that the VLP-HPV 16 E7 fusion or insertion site is not important. In addition, the results confirm the findings of the previous challenge (Figure 3) and show strong efficacy against well-established tumors ranging in size from 0.5 to 0.9 cm^3^.

### Therapeutic Potency of the VLP-E7 CIN-cervical Cancer Candidate Vaccine

**Figure 5:**
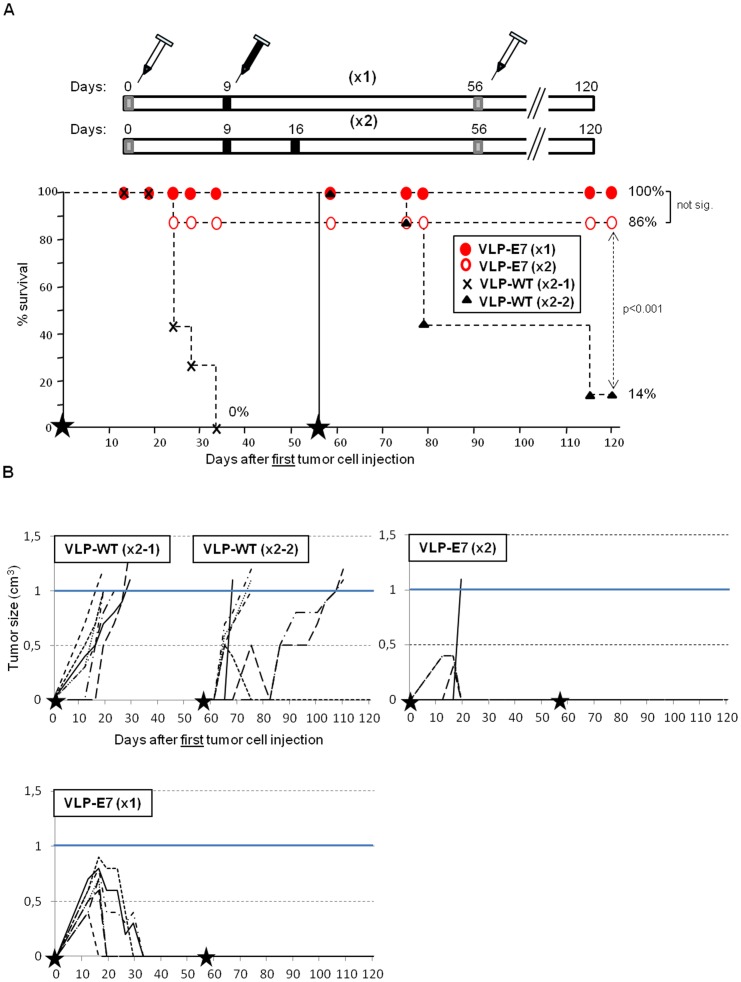
Therapeutic tumor challenge III. **A)** Top, vaccination schedule. Bottom, tumor survival data (n = 7). Black stars correspond to days 0 and +56, when tumor cells (5×10^5^/mouse) were inoculated. Mice vaccinated once (×1, day +9) with VLP-E7 are represented with red (closed) circles. The remaining symbols correspond to the vaccinated mice as indicated in the square in the survival diagram. **B)** Evolution of tumor size. Values shown are from individual mice in each group, as indicated in each panel. Animals were sacrificed when tumors reached 1 cm^3^ (blue line). Black stars indicate subcutaneous tumor cell injection days (0 and +56).

To evaluate the therapeutic potency/efficacy of the VLP-E7 vaccine, we designed the experiment shown in Figure 5A. Mice (n = 7/group) were vaccinated with VLP-E7 with either one dose (group ×1) or two doses (group ×2) at days +9 or +9 and +16 post-inoculation, respectively. The two control groups were also vaccinated twice (VLP-WT ×2-1 and -2). However, only the VLP-WT ×2-1 group received the first tumor cell injection, whereas the VLP-WT ×2-2 group was reserved as VLP-vaccinated and age-matched controls for the second tumor cells inoculation.

All the mice had developed tumors by the day of the first vaccination. Interestingly, 50 days after inoculation, mice vaccinated with one or two doses of VLP-E7 were almost all alive (100% [7/7] and 86% [6/7], respectively). In contrast, all control mice (VLP-WT ×2-2) died before day 30 (Figure 5A and 5B). Remarkably, mice vaccinated with one dose of VLP-E7 were able to eradicate established large tumors (0.5 to 0.9 cm^3^). Mice vaccinated twice with VLP-E7 were also able to eliminate tumors of 0.4–0.6 cm^3^ (see Figure 5B), thus confirming our previous results (Figure 4).

To evaluate the long-lasting anti-tumor immune response, the mice that remained alive in the VLP-E7 ×1 and VLP-E7 ×2 groups (13 out of 14 in total) were re-challenged with a second tumor cell inoculation. VLP-WT ×2–2 group of mice were the age-matched and VLP-vaccinated control mice. Importantly, 64 days after the second inoculation, all of the VLP-E7–vaccinated mice (7 from group ×1 and 6 from group ×2) were tumor-free and alive. In contrast, 6 out of 7 control mice died (14% survival) (Figure 5A and 5B).

Our findings show that a single subcutaneous injection of VLP-E7 without adjuvant was effective, as it eradicated established large tumors (0.4 to 0.9 cm^3^) in 100% of mice, with tumor-free survival after a four-month observation period and after two very aggressive inoculations.

## Discussion

We analyzed the therapeutic efficacy of a CIN-cervical cancer candidate vaccine, VLP-E7, using IBDV VLPs with T1 symmetry as a carrier. Vaccines are thought to become an important alternative in cancer therapy and are currently used in combination with traditional cancer therapies (radiotherapy, chemotherapy, and surgery) [17]. The development of effective therapeutic cancer vaccines has proven extremely challenging. In the last few decades, several techniques using different carriers (plasmid DNA, viruses, peptides) have achieved impressive results in pre-clinical (eg, murine) studies. However, much work remains to be done in human trials [17], [29].

The key aspects in a human vaccine are its safety and efficacy profile and the use of approved adjuvants with mild or no side effects. VLPs have proven to be an effective carrier, capable of inducing a strong immune response. Moreover, their lack of genetic material means that they carry no biological risk. The results of IBDV VLPs used as a vaccine for IBDV treatment are excellent. However, there are no data from pre-clinical studies or human trials using IBDV as treatment for human diseases.

We used IBDV VLPs with T1 symmetry as the carrier of HPV-16 E7 epitopes. IBDV VLP elicits strong immune responses for a series of reasons: i) **Size**. Particles ranging in size from 20 to 100 nm are efficiently uptaken by DCs. The IBDV VLP-WT and VLP-E7 are 25 and 45 nm in size, respectively. Our binding results confirm this aspect (see Figure S3), and suggest the existence of IBDV-VLP specific receptors on the DC surface. In this sense a VLA-4 integrin binding motif has been described in the IBDV-VP2 protein [30], supporting again our data. ii) **Structure**. Repetitive surfaces and particulate structures are ideal for obtaining both a good antibody response and MHC cross-presentation. IBDV VLPs with T1 symmetry fit in this category. iii) **DC maturation**. IBDV VLPs are able to bind and activate human DCs and murine DCs (see Figures S1B and S1C, and Figure S3B). Similar behavior has been described for the HPV-VLP [31]. iv) **Targets other than DC**. IBDV VLPs infect both chicken IgM B cells and macrophages [32], suggesting a co-stimulatory effect and DC maturation. If this effect is also documented in mice, it could explain the strong anti-tumor response observed in our study. The same co-stimulatory effect could be possible in humans. v) **Ability to display foreign disease-related epitopes**. IBDV VLPs can display heterologous sequences of up to 60 aa in different VLP positions (A.G., J.J.B., T.Z., and C.V.K., present work and unpublished results). This feature is critical if a specific immune response is to be obtained.

Combining the strong immune actions of IBDV VLPs with the specificity of HPV-16 E7 sequences enabled us to generate chimeric VLPs by genetic fusion (VLP-E7) for the treatment of established HPV-16–related tumors. Our results support the idea of IBDV VLPs as efficient carriers capable of eliciting a specific and strong immune response against large established HPV-16 E7 epitope–bearing tumors in mice.

The model we applied (HLA-A2 humanized transgenic C57BL/6 mice and TC1/A2 tumor cells expressing HPV-16 E6/E7 antigens) has been widely used for testing anti-cervical cancer vaccines [21].

We demonstrated that immunization with VLP-E7 induces IFN- γ –secreting splenocytes against HPV-related T-cell epitopes in the absence of adjuvants (ELISPOT assay). We do not know why the presence of adjuvants did not boost the effect of VLP-E7. One possibility would be that the presence of some adjuvants could break the virus-like structure, thus decreasing its immunogenicity. However, this drawback could be overcome by injecting the VLP and adjuvants separately.

Other possibility is that the endogenous co-adjuvant activity of IBDV-VLP (see Figure S1) is *per se* strong enough that is not boost by any of the classical adjuvants tested. However more studies will be necessary to investigate this activity in more detail.

The most relevant results came from the *in vivo* therapeutic tumor challenge experiments. VLP-E7 vaccination at days +5 and +12 after inoculation protected 100% of the animals from tumor development and some of the animals from regression of small tumors (0.2–0.3 cm^3^). In contrast, all control mice died. This result confirmed a previous one where we were able to completely protect the mice, although the tumor was induced with fewer tumor cells and not all the mice developed tumors (data not shown). The next step was to test the VLP-E7 vaccine in a more stringent situation, that is, against tumors larger than 0.2–0.3 cm^3^. Delaying the vaccination schedule 4 days (+9 and +16) revealed the construct to be highly efficacious: it eradicated well-established tumors larger than 0.5 cm^3^ with 88.8% survival (0% in control mice). This new timing (+9 and +16 post-inoculation) illustrated the optimal schedule for the evaluation of the therapeutic efficacy of the vaccines. In this scenario, some animals developed tumors close to the maximum ethically permitted volume of 1 cm^3^.

Lastly, we demonstrated the strength of the immune response brought about by the VLP-E7 vaccine under severely adverse conditions, not only against large established tumors and without adjuvant, but also against a second tumor challenge and testing with only one vaccination (day +9 after the first tumor induction). The results obtained were excellent, with 100% protection and tumor-free survival after a four-month observation period.

There is no standardized therapeutic cancer vaccine on the market. The treatment of well-established tumors is a considerable challenge, and several groups have reported therapeutic activity with CIN-cervical cancer candidate vaccines [33], [34], [35], [36]. Only a few authors have reported regressions of tumors above 0.5 cm^3^ [20], [37], in addition to efficacy against two inoculations (re-challenge), as we observed in our study. Other cervical cancer candidate vaccines, mainly those based on peptides or fusion proteins, need several injections (over 4–5) and high amounts of immunogen (up to 500 µg/injection) to achieve anti-tumor responses [3]. In addition, the use of adjuvants proved necessary in most published studies. Our data were obtained without adjuvants and together the *in vitro* DC maturation experiments (Figure S1) strongly suggest that our IBDV-VLP–based platform is able, *per se*, to activate immune cells, again supporting its role as an excellent carrier. The use of adjuvants is an important safety issue in human clinical studies. To date, only three adjuvants have been approved for human use, namely, aluminium hydroxide (Alum), oil-in-water emulsions, and AS04 [25].

During the course of the experiments shown here, we tested our candidate vaccine VLP-E7 against other vaccines displaying the E7 epitope in different structures and/or conformations. As explained above, one of the current approaches for CIN-cervical cancer vaccines is the use of naked peptides. We compared the *in vivo* efficacy of VLP-E7 with a peptide containing the same HPV-16 E7 sequence (five-fold molar excess compared to VLP-E7). The results clearly indicate that the VLP-E7 candidate is more efficacious than the E7 peptide (100% *vs* 30% efficacy) (Figure 3). Nevertheless, these experiments were performed in an adjuvant-free setting, and it remains to be determined whether adjuvants could have improved their efficacy. We also compared VLP-E7 with a new vaccine, in which the E7 region was cloned in a different position (VLP-E7-B). The *in vivo* results revealed no significant differences (p>0.05) between the two candidates, provided excellent tumor-free and survival data (77% to 88%).

The magnitude and duration of the immune response required for the clearance of established tumors are critical aspects in the efficacy of therapeutic cancer vaccines. The results presented in this report show that the VLP-E7 candidate fulfills these requirements: i) remarkable efficacy against well-established large tumors (in the last challenge 5 mice eradicated tumors measuring 0.7–0.9 cm^3^ and a further 5 eradicated tumors measuring 0.4–0.6 cm^3^ [Figure 5]); and ii) long-lasting anti-tumor response against 2 inoculations, with 100% protection after 4 months of treatment (4 months in mice corresponds to about 10% of their life span, equivalent to 7–8 years in humans).

In summary, our results highlight the immunogenic potential and *in vivo* efficacy of the VLP-E7 candidate, showing it to be a promising vaccine for the therapy of CIN lesions and cervical cancer. Further studies will be necessary to investigate the potential of the IBDV VLP carrier and its applications to other human diseases.

## Supporting Information

Figure S1 *In vitro* maturation of DCs. **A)** Scheme of the differentiation and maturation process. For differentiation into immature DCs, murine monocytes were incubated only with GM-CSF, whereas human monocytes were incubated with both GM-CSF and IL-4. The surface markers used in this study are shown in the figure (h; human, m; murine). **B)** Human DC activation. After incubation with either PBS alone (control), cytokine cocktail (positive control) or IBDV-VLP, the immunophenotype of the cells was analyzed by FACS. X and Y-axes represent CD80 and CD86 expression, respectively. The percentages of mature hDCs are shown in each quadrant. **C)** Murine DC activation. After incubation with either PBS alone (control), LPS (positive control) or IBDV-VLP (2 µg/ml and 0.5 µg/ml), the immunophenotype of the cells was analyzed by FACS. **Right**; Dot plots allowing the detection of viable and dendritic cells (gated as CD11c^+^ MHC II^+^ cells), of two representative samples (control and LPS treated). As shown, around 90% of the cells were CD11c+. **Left**; Expression levels of the indicated maturation markers of each sample.
(TIF)

Figure S2 IL-12 p70 and IL-6 production. Production of cytokines was analyzed by ELISA after stimulation of immature DCs with either 5 µg/ml LPS, IBDV-VLP (2 µg/ml and 0.5 µg/ml) or PBS alone (control). Bars represent mean ± s.e.m. of triplicates.
(TIF)

Figure S3 Binding of the IBDV-VLPs to immature hDCs. **A)** Scheme of the rationale of the binding assay. **B)** Histograms show the binding of IBDV-VLP on the immature hDC surface. Negative control (background signal) corresponds to the cells incubated with the Cy5-conjugated mAb anti-VLP.
(TIF)

Methods S1 Chimeric Infectious Bursal Disease Virus-Like Particles as Potent Vaccines for Eradication of Established HPV-16 E7–dependent Tumors.
(DOC)
