# Supplementary figures and images for: Chimeric Infectious Bursal Disease Virus-Like Particles as Potent Vaccines for Eradication of Established HPV-16 E7–Dependent Tumors
Source: PLoS One. 2012 Dec 31;7(12):e52976. doi: 10.1371/journal.pone.0052976 (PMC3534127; doi:10.1371/journal.pone.0052976)

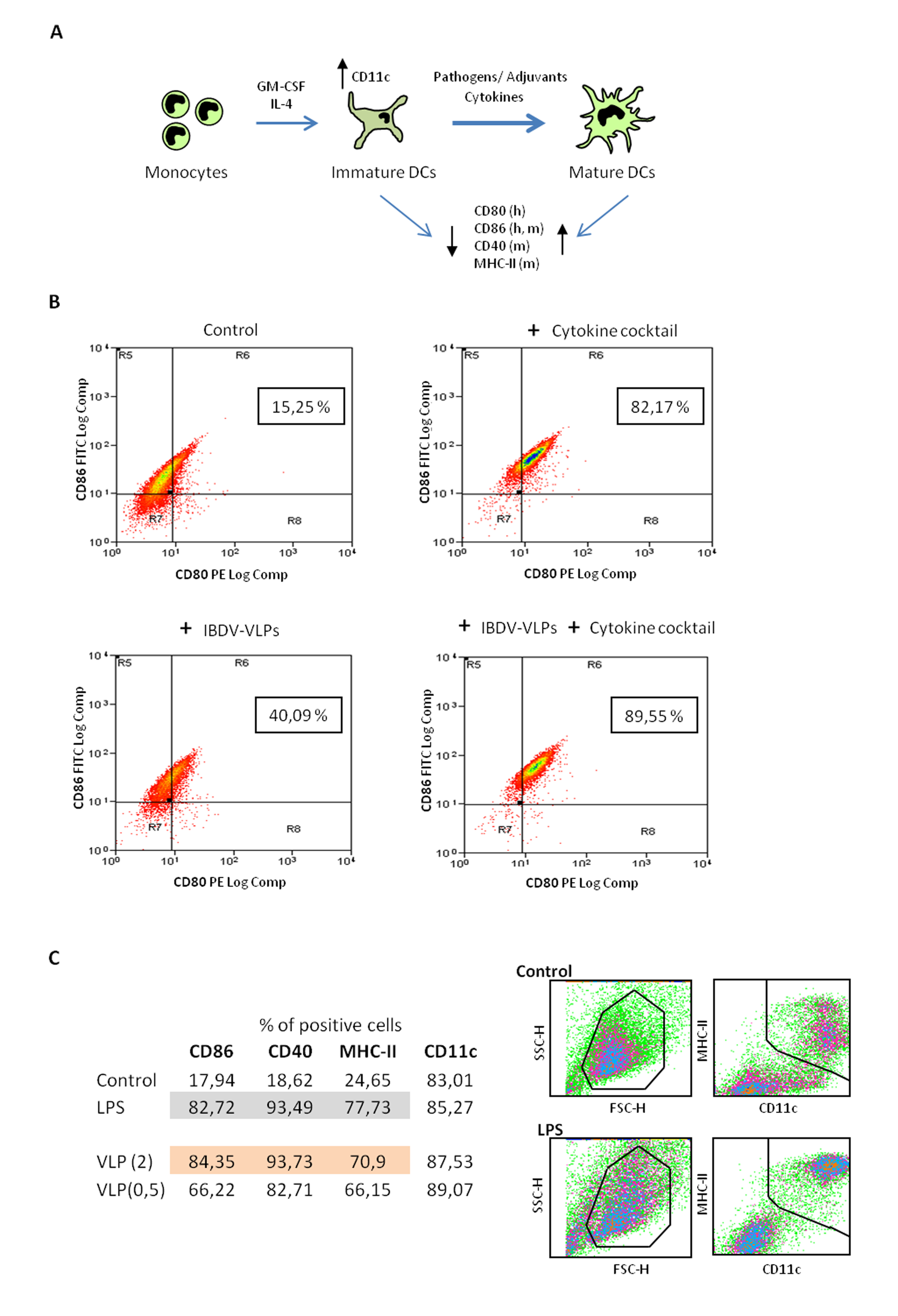

Supplement: Figure S1 — In vitro maturation of DCs. A) Scheme of the differentiation and maturation process. For differentiation into immature DCs, murine monocytes were incubated only with GM-CSF, whereas human monocytes were incubated with both GM-CSF and IL-4. The surface markers used in this study are shown in the figure (h; human, m; murine). B) Human DC activation. After incubation with either PBS alone (control), cytokine cocktail (positive control) or IBDV-VLP, the immunophenotype of the cells was analyzed by FACS. X and Y-axes represent CD80 and CD86 expression, respectively. The percentages of mature hDCs are shown in each quadrant. C) Murine DC activation. After incubation with either PBS alone (control), LPS (positive control) or IBDV-VLP (2 µg/ml and 0.5 µg/ml), the immunophenotype of the cells was analyzed by FACS. Right; Dot plots allowing the detection of viable and dendritic cells (gated as CD11c+ MHC II+ cells), of two representative samples (control and LPS treated). As shown, around 90% of the cells were CD11c+. Left; Expression levels of the indicated maturation markers of each sample. (TIF) [file pone.0052976.s001.tif]

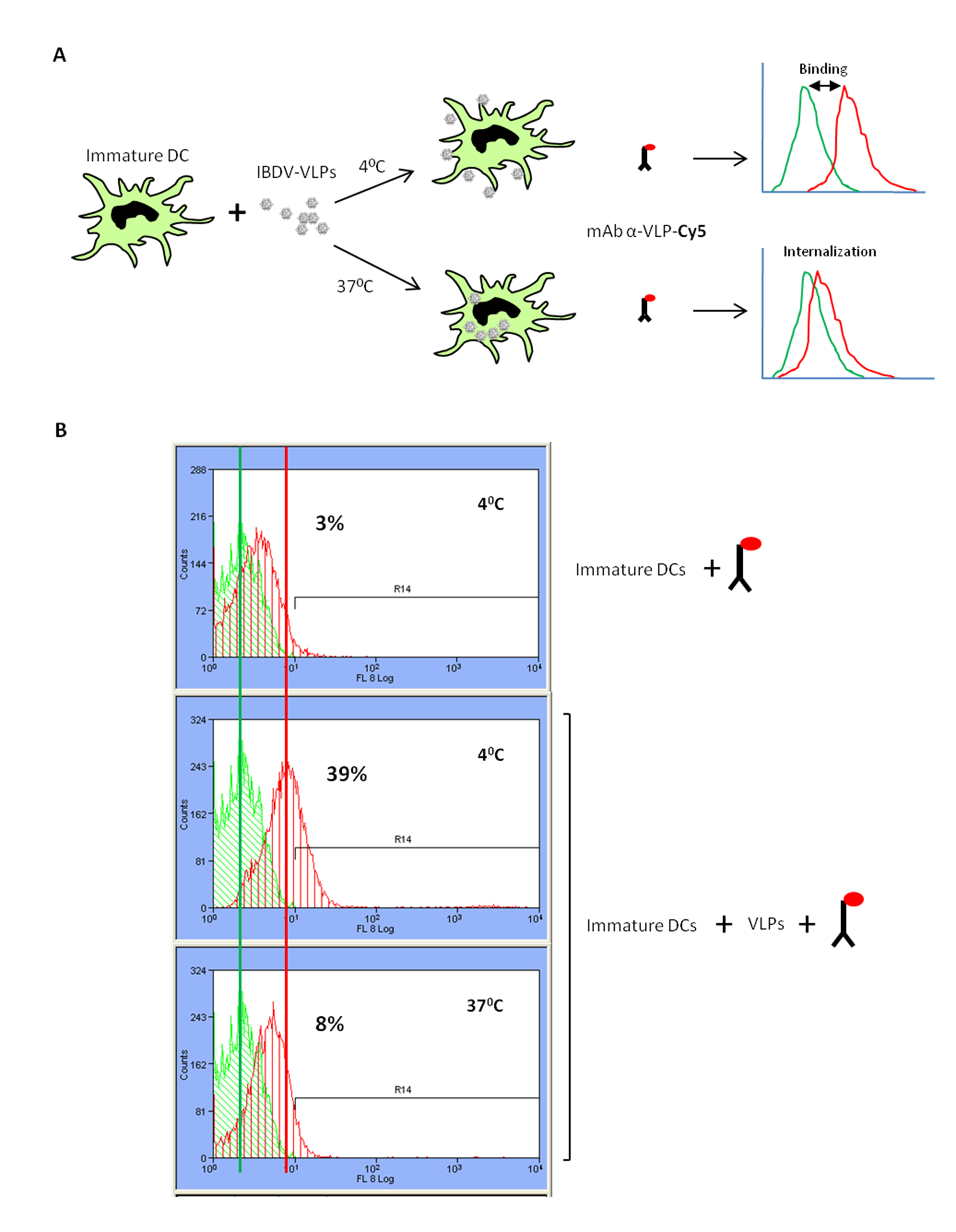

Supplement: Figure S3 — Binding of the IBDV-VLPs to immature hDCs. A) Scheme of the rationale of the binding assay. B) Histograms show the binding of IBDV-VLP on the immature hDC surface. Negative control (background signal) corresponds to the cells incubated with the Cy5-conjugated mAb anti-VLP. (TIF) [file pone.0052976.s003.tif]
