# Supplementary material for: Chimeric Infectious Bursal Disease Virus-Like Particles as Potent Vaccines for Eradication of Established HPV-16 E7–Dependent Tumors
Source: PLoS One. 2012 Dec 31;7(12):e52976. doi: 10.1371/journal.pone.0052976 (PMC3534127; doi:10.1371/journal.pone.0052976)
Supplement: Methods S1 — Chimeric Infectious Bursal Disease Virus-Like Particles as Potent Vaccines for Eradication of Established HPV-16 E7–dependent Tumors. (DOC) [file pone.0052976.s004.doc]

**SUPPORTING INFORMATION**

**Chimeric Infectious Bursal Disease Virus-Like Particles as Potent Vaccines for Eradication of Established HPV-16 E7–dependent Tumors**

Juan Martin Caballero ^1, *^, Ana Garzón ^2, *^, Leticia González-Cintado ^3^, Wioleta Kowalczyk ^4^, Ignacio Jimenez Torres ^2^, Gloria Calderita ^2^, Margarita Rodriguez ^2^, Virgínia Gondar ^2^, Juan Jose Bernal ^2^, Carlos Ardavín ^3^, David Andreu ^4^, Thomas Zürcher ^2^, Cayetano von Kobbe ^2, ¶^

^1^  Laboratory Animal Unit, Barcelona Biomedical Research Park, Barcelona, Spain

^2^  Cancer Vaccines Unit, R & D Department, Chimera Pharma S.L.U., Madrid, Spain

^3^ Department of Immunology and Oncology, Centro Nacional de Biotecnología/CSIC, Madrid, Spain.

^4^ Department of Experimental and Health Sciences, Pompeu Fabra University, Barcelona Biomedical Research Park, Barcelona, Spain

*These authors contributed equally to this work.

^¶^ *Corresponding author*:

Cayetano von Kobbe, Molecular Biology Centre “Severo Ochoa”, Spanish Research Council (CBMSO-CSIC), Universidad Autónoma de Madrid, Campus de Cantoblanco, Madrid, Spain (former Chimera-Pharma´s Cancer Vaccines Unit group leader);

Tel.: 34-91-196 4651; e-mail: [cvonkobbe@cbm.uam.es](mailto:cvonkobbe@cbm.uam.es)

**SUPPLEMENTAL METHODS**

*Monocyte isolation and in vitro DC differentiation and activation*

Murine monocytes were isolated as described [1]. Differentiation of monocytes into DCs was achieved by culturing the cells in RPMI 1640 medium supplemented with 10% FCS and 20 ng/ml Gm-CSF (Peprotech, London, UK) for 48 h. at 37^0^C and 5% CO_2_. Activation of DCs was achieved after treatment with either 5 µg/ml LPS from *E. Coli* (Sigma, St. Louis, Mo, USA) or baculovirus-purified IBDV-VLP (2 µg/ml and 0.5 µg/ml) for 16 h., at 150000 cells/well. The phenotypic analysis of mature DCs was performed after triple staining with FITC-conjugated anti-MHC II, allophycocyanin (APC)-conjugated anti-CD11c, and biotin-conjugated anti-CD86 or anti-CD40, followed by streptavidin-PerCP. Human monocytes were obtained from healthy donor peripheral blood from buffy coats. The PBMCs were isolated by standard Ficoll (Lymphoprep^TM^) density gradient centrifugation. After immunomagnetic depletion, monocyte preparations were differentiated to immature DCs in X-VIVO medium (Bio Whittaker, Cambrex) supplemented with 1000 U/ml IL-4, 50 ng/ml GM-CSF (Peprotech, London, UK) and 1% AB serum (Sigma). To obtain mature DCs, either a cytokine cocktail (1000 U/ml IL-4, 90 ng/ml GM-CSF, 10 ng/ml IL-1β, 10 ng/ml TNF-α, 1000 U/ml Il-6, 10 ng/ml PGE2 and 1 µg/ml LPS)(Peprotech, London, UK, except LPS that was from Sigma), IBDV-VLP (1 µg/10^6^ immature DC) or PBS alone (control) was added for 16 h. The immunophenotype of immature and mature DCs was assessed by FACS analysis using FITC-conjugated mAb anti-CD86 and PE-conjugated mAb anti-CD (BD Biosciences).

*Cytokine production*

Production of IL-12p70 and IL-6 was analyzed 16 h. after stimulation of 150000 immature mDCs in 24-well plates, with either 0,5 µg/ml LPS, baculovirus-purified IBDV-VLP (2 µg/ml and 0.5 µg/ml) or PBS alone (control). IL-12 and IL-6 were detected using BD OPtEIA ELISA kits (BD Biosciences).

*Binding assays*

Immature hDCs were obtained as indicated before, and then incubated with IBDV-VLPs (10 µg VLP/10^6^ DCs) either at 4^0^C or 37^0^C for 1 h. To detect the VLPs on the cell surface, a Cy5-conjugated mAb anti-VLP was added and then analyzed by FACS. As negative control, the cells were incubated with the Cy5-conjugated mAb anti-VLP alone.

**SUPPLEMENTAL BIBLIOGRAPHY**

1. Dominguez PM, Lopez-Bravo M, Kalinke U, Ardavin C (2011) Statins inhibit iNOS-mediated microbicidal potential of activated monocyte-derived dendritic cells by an IFN-beta-dependent mechanism. Eur. J. Immunol. 41: 3330-3339
